# Bioinformatic and Molecular Analysis of Inverse Autotransporters from Escherichia coli

**DOI:** 10.1128/mSphere.00572-19

**Published:** 2019-08-28

**Authors:** Kelvin G. K. Goh, Danilo G. Moriel, Steven J. Hancock, Minh-Duy Phan, Mark A. Schembri

**Affiliations:** a School of Chemistry and Molecular Biosciences, University of Queensland, Brisbane, Queensland, Australia; b Australian Infectious Diseases Research Centre, University of Queensland, Brisbane, Queensland, Australia; University of Kentucky

**Keywords:** *Escherichia coli*, autotransporter proteins, biofilms

## Abstract

Proteins secreted by the type V secretion system possess multiple functions, including the capacity to mediate adhesion, aggregation, and biolfilm formation. The type V secretion system can be divided into five subclasses, one of which is the type Ve system. Proteins of the type Ve secretion system are also referred to as inverse autotransporters (IATs). In this study, we performed an *in silico* analysis of 126 completely sequenced Escherichia coli genomes available in the NCBI database and identified several distinct IAT-encoding gene families whose distribution varied throughout the E. coli phylogeny. The genes included three characterized IATs (intimin, *fdeC*, and *yeeJ*) and four uncharacterized IATs (here named *iatA*, *iatB*, *iatC*, and *iatD*). The four *iat* genes were cloned from the completely sequenced environmental E. coli strain SMS-3-5 and characterized. Three of these IAT proteins (IatB, IatC, and IatD) were expressed at the cell surface and possessed the capacity to mediate biofilm formation in a recombinant E. coli K-12 strain. Further analysis of the *iatB* gene, which showed a unique association with extraintestinal E. coli strains, suggested that its regulation is controlled by the LeuO global regulator. Overall, this study provides new data describing the prevalence, sequence variation, domain structure, function, and regulation of IATs found in E. coli.

**IMPORTANCE** Escherichia coli is one of the most prevalent facultative anaerobes of the human gut. E. coli normally exists as a harmless commensal but can also cause disease following the acquisition of genes that enhance its pathogenicity. Adhesion is an important first step in colonization of the host and is mediated by an array of cell surface components. In E. coli, these include a family of adhesins secreted by the type V secretion system. Here, we identified and characterized new proteins from an emerging subclass of the type V secretion system known as the inverse autotransporters (IATs). We found that IAT-encoding genes are present in a wide range of strains and showed that three novel IATs were localized on the E. coli cell surface and mediated biofilm formation. Overall, this study provides new insight into the prevalence, function, and regulation of IATs in E. coli.

## Abstract (precis)

Escherichia coli is one of the most prevalent facultative anaerobes of the human gut. E. coli normally exists as a harmless commensal but can also cause disease following the acquisition of genes that enhance its pathogenicity. Adhesion is an important first step in colonization of the host and is mediated by an array of cell surface components. In E. coli, these include a family of adhesins secreted by the type V secretion system. Here, we identified and characterized new proteins from an emerging subclass of the type V secretion system known as the inverse autotransporters (IATs). We found that IAT-encoding genes are present in a wide range of strains and showed that three novel IATs were localized on the E. coli cell surface and mediated biofilm formation. Overall, this study provides new insight into the prevalence, function, and regulation of IATs in E. coli.

## INTRODUCTION

Escherichia coli is one of the most prevalent facultative anaerobes of the human gut and harbors genes encoding a wide array of surface-expressed factors that promote the colonization of specific niches. One such factor includes the highly abundant group of proteins secreted by the type V secretion system (1, 2). All proteins secreted by this system share several common features: (i) an N-terminal signal sequence that targets the protein to the Sec machinery for transport across the inner membrane, (ii) a passenger domain that is either cell surface exposed or secreted, and (iii) a translocator (or β-barrel) domain that is embedded in the outer membrane and helps to facilitate the translocation of the passenger domain (2–5). The passenger domain of these proteins determines the unique functional characteristics of an individual protein. Overall, proteins secreted by the type V system possess a wide range of functions, including adhesion, cell-to-cell aggregation, and biofilm formation (6–8), as well as protease and cytotoxic activity (9, 10). For example, the well-characterized autotransporter (AT) protein antigen 43 (Ag43) of uropathogenic E. coli (UPEC) contributes to adhesion, cell-to-cell aggregation, biofilm formation, and long-term persistence in the urinary tract (11–14).

AT proteins can be classified into five subclasses, namely, types Va (monomeric AT), Vb (two-partner secretion system), Vc (trimeric), Vd (fused two-partner secretion system), and Ve (inverse ATs [IATs]) (1, 15, 16). The domain organization of IATs resembles that of the classical type Va AT proteins but with the passenger and translocation domains in opposite locations within the primary amino acid sequence. Two well-studied proteins from the type Ve subclass include intimin from E. coli and invasin from enteropathogenic *Yersinia* species (17–19). Intimin is an adhesin expressed by enteropathogenic E. coli (EPEC) and enterohemorrhagic E. coli (EHEC) and contributes to the formation of actin pedestals leading to attaching and effacing lesions in the gut (20, 21). Invasin-mediated adherence of enteropathogenic *Yersinia* to host cells triggers the envelopment of bacterial cells through host cell-mediated autophagy and plays an early role in the infection cycle by binding directly to host β_1_-integrins (22, 23).

The enormous volume of data available from genome sequencing has facilitated the identification of IATs from different phyla of bacteria (17, 24). However, many IATs still remain to be identified and characterized. In this study, we sought to identify the complement of IAT proteins found in E. coli and to characterize their phenotypic properties. To this end, we first probed 126 completely sequenced E. coli genomes available in the NCBI database for the presence of genes encoding IAT proteins. Next, we cloned, expressed, and characterized the function of three new IAT proteins, one of which was also examined at the regulatory level. Overall, this study has defined the set of IAT proteins found in E. coli.

## RESULTS

### E. coli possesses a diverse range of IAT genes.

The β-barrel domain represents the most conserved region of IAT proteins, and the presence of an intimin-like β-barrel domain defines the IAT family (18, 24). As such, we used the β-barrel amino acid sequences of intimin and invasin to probe a database of annotated protein sequences from 126 completely sequenced E. coli genomes (see Data Set S1 in the supplemental material). The subsequent list of hits represented proteins that contained the IAT β-barrel Pfam domain (PF11924). The amino acid sequences of these β-barrel domains were aligned, revealing seven distinct groups of IAT proteins (Fig. S1). Among these, intimin (encoded by the *eaeA* gene) has been very well characterized and thus was not examined further. The remaining IATs, the genes for which were all present in the environmental E. coli strain SMS-3-5, were selected for further study (Table 1). The six IAT genes from SMS-3-5 include the previously studied *fdeC* and *yeeJ* genes (25–27) and four uncharacterized IAT genes found at different locations on the chromosome and renamed as follows: EcSMS35_1920 (*iatA*), EcSMS35_2661 (*iatB*), EcSMS35_4024 (*iatC*), and EcSMS35_4876 (*iatD*).

**TABLE 1:** E. coli SMS-3-5 type Ve AT proteins

Locus tag	GenBank accession no.	Gene	Gene size (bp)	Protein size (aa)^*a*^
EcSMS35_0331	ACB16013.1	*fdeC*	4,245	1,415
EcSMS35_1146	ACB16711.1	*yeeJ*	7,077	2,359
EcSMS35_1920	ACB19099.1	*iatA*	1,395	465
EcSMS35_2661	ACB17431.1	*iatB*	2,175	725
EcSMS35_4024	ACB17037.1	*iatC*	8,802	2,934
EcSMS35_4876	ACB20062.1	*iatD*	5,241	1,747

a aa, amino acids.

10.1128/mSphere.00572-19.1 FIG S1 Cladogram of the β-barrel domain from putative IAT proteins found in 126 complete E. coli genomic sequences. A database of annotated proteins from each strain was probed using fasta36 to identify putative β-barrel domain-containing IAT proteins. The context of the β-barrel domain was examined in each extracted protein sequence to ensure that it was located at the N-terminal end of the protein. The six prototype putative proteins chosen from SMS-3-5 are highlighted in red, and the branch representing intimin is in gray. Alignments and phylogenetic trees were drawn in MEGA7 and visualized with FigTree. Download FIG S1, PDF file, 0.2 MB. Copyright © 2019 Goh et al. 2019 Goh et al. This content is distributed under the terms of the Creative Commons Attribution 4.0 International license.

10.1128/mSphere.00572-19.5 DATA SET S1 Prevalence of IAT proteins in the collection of 126 completely sequenced E. coli genomes. Download Data Set S1, XLSX file, 0.1 MB. Copyright © 2019 Goh et al. 2019 Goh et al. This content is distributed under the terms of the Creative Commons Attribution 4.0 International license.

### Analysis of the six IAT genes in E. coli.

**FIG 1:**
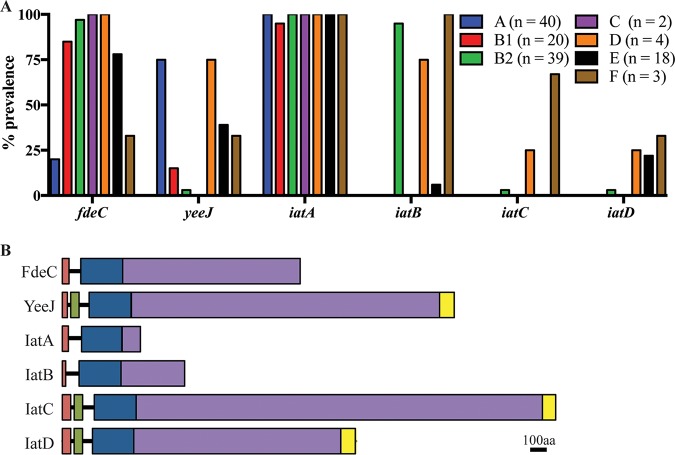
(A) Prevalence of IAT-encoding genes within phylogroups A (*n* = 40), B1 (*n* = 20), B2 (*n* = 39), C (*n* = 2), D (*n* = 4), E (*n* = 18), and F (*n* = 3). (B) Schematic diagram of E. coli SMS-3-5 IAT proteins. Predicted domains are shown as colored boxes (orange, signal sequence; green, LysM domain; blue, β-barrel domain; purple, passenger domain; yellow, C-type lectin domain). aa, amino acids.

The prevalence of the six IAT genes was assessed in the 126 completely sequenced E. coli genomes. A complete gene was found in 67% of strains for *fdeC* (84/126), 35% for *yeeJ* (44/126), 99% for *iatA* (124/126), 35% for *iatB* (44/126), 3% for *iatC* (4/126), and 6% for *iatD* (7/126). A breakdown of the prevalence of the genes within each phylogroup is depicted in Fig. 1A. To extend this analysis, we examined the well-defined E. coli Reference (ECOR) collection of 72 strains using a PCR screening approach. The ECOR collection comprises strains isolated from a variety of hosts and locations and is representative of the ecological and phylogenetic diversity of the E. coli species. The correct-sized PCR product was found in 83% of strains for the *fdeC* gene, 36% for *yeeJ*, 90% for *iatA*, 36% for *iatB*, 19% for *iatC*, and 10% for *iatD* (Data Set S2).

10.1128/mSphere.00572-19.6 DATA SET S2 Prevalence of *iat* genes in the ECOR collection. Download Data Set S2, XLSX file, 0.1 MB. Copyright © 2019 Goh et al. 2019 Goh et al. This content is distributed under the terms of the Creative Commons Attribution 4.0 International license.

Next, we examined the genomic context of each of the E. coli IAT genes. The six IAT genes occur at different chromosomal locations; note that the position of each orthologue is conserved in all of the strains examined (Fig. S2). Both *iatA* and *iatD* were highly conserved (amino acid identity of >98%). In contrast, the *iatB* and *iatC* genes varied in size, with the predicted IatB protein ranging from 725 to 743 amino acids (amino acid identity of >89%) and the predicted IatC protein ranging from 2,459 to 2,965 amino acids (amino acid identity of >78%). IatB and IatC possessed a highly conserved signal sequence and translocation domain, respectively, with their variability attributed solely to sequence changes in the passenger domain.

10.1128/mSphere.00572-19.2 FIG S2 Genomic context of the IAT genes (highlighted in red) in SMS-3-5. Depicted are two genes upstream and downstream of the IAT gene of interest and the location of the fragment on the genome of SMS-3-5. Genes with known functions are labeled with gene names, and genes of unknown function are labeled with locus tags. Download FIG S2, PDF file, 0.2 MB. Copyright © 2019 Goh et al. 2019 Goh et al. This content is distributed under the terms of the Creative Commons Attribution 4.0 International license.

### The six IAT genes from SMS-3-5 encode proteins with similar domain organizations.

A schematic diagram representing the domain organizations of the six IAT proteins from SMS-3-5 is shown in Fig. 1B. The signal sequence of each protein is followed by an IAT β-barrel Pfam domain (PF11924). Modeling of the β-barrel domains from all six proteins revealed that they are very similar in size (243 to 246 amino acids) and are predicted to fold into a 12-stranded β-barrel structure. Overall, the β-barrel domains of these proteins share 34 to 88% amino acid identity. A comparative amino acid alignment against the β-barrel sequences from subtype Va AT proteins (Ag43, UpaH, Sat, and Vat) revealed that the sequences of each subgroup cluster independently and are highly variable between the different subgroups of AT proteins (Fig. S3).

10.1128/mSphere.00572-19.3 FIG S3 Comparison of β-barrel amino acid sequences from IAT and type Va AT proteins. (Left) Cladogram generated using the β-barrel amino acid sequences from IAT and type Va AT proteins. The sequences from the different subgroups cluster separately. (Right) Pairwise comparison of each β-barrel sequence. Numbers represent the percent identity. Download FIG S3, PDF file, 0.2 MB. Copyright © 2019 Goh et al. 2019 Goh et al. This content is distributed under the terms of the Creative Commons Attribution 4.0 International license.

Analysis of the C-terminal passenger domain of each IAT on InterPro revealed that FdeC, YeeJ, IatC, and IatD contain various numbers of Big1 repeats, which are typical of IATs. Nonetheless, structural modeling of the passenger domains of IatA and IatB on Phyre2 suggests that they possess structural characteristics (e.g., a fibronectin III domain) similar to those of other bacterial immunoglobulin superfamily (IgSF) domains. The passenger domains of the three larger proteins (YeeJ, IatC, and IatD) are capped with a C-type lectin domain. Additionally, a LysM domain is present at the N-terminal end of the β-barrel domain of these three larger proteins.

### Cloning and expression of IAT genes from SMS-3-5.

In order to examine the functional properties of the four uncharacterized IAT proteins, the *iatA*, *iatB*, *iatC*, and *iatD* genes were amplified from SMS-3-5 and cloned into the isopropyl-β-d-1-thiogalactopyranoside (IPTG)-inducible pSU2718 expression vector to generate plasmids pIatA, pIatB, pIatC, and pIatD, respectively. A list of strains and plasmids used is provided in Data Set S3. The plasmids were transformed into the E. coli K-12 *flu* deletion strain MS427, which is unable to mediate cell aggregation and biofilm formation normally associated with Ag43 expression (28). Specific antisera were generated against the C-terminal passenger domains of IatB, IatC, and IatD and used to confirm their expression by Western blot analysis of whole-cell lysates prepared from IPTG-induced cultures grown overnight (Fig. S4). These experiments resulted in the detection of bands corresponding to full-length IatB, IatC, and IatD proteins as well as lower-molecular-weight bands presumed to be breakdown products based on their specific antibody cross-reactivity. Despite our efforts, we were unable to generate an IatA antiserum of sufficient quality and could not reliably detect the expression of the IatA protein. Hence, only IatB, IatC, and IatD were further characterized.

10.1128/mSphere.00572-19.4 FIG S4 Western blot analysis of the different IAT proteins. For each blot, − represents whole-cell lysates prepared from the MS427(pSU2718) vector control, and + represents whole-cell lysates prepared from MS427 expressing the different IAT proteins, IatB (81 kDa) (A), IatC (307 kDa) (B), and IatD (188 kDa) (C). Download FIG S4, PDF file, 0.1 MB. Copyright © 2019 Goh et al. 2019 Goh et al. This content is distributed under the terms of the Creative Commons Attribution 4.0 International license.

10.1128/mSphere.00572-19.7 DATA SET S3 Strains and plasmids used in this study. Download Data Set S3, XLSX file, 0.1 MB. Copyright © 2019 Goh et al. 2019 Goh et al. This content is distributed under the terms of the Creative Commons Attribution 4.0 International license.

### IatB, IatC, and IatD are located on the cell surface.

**FIG 2:**
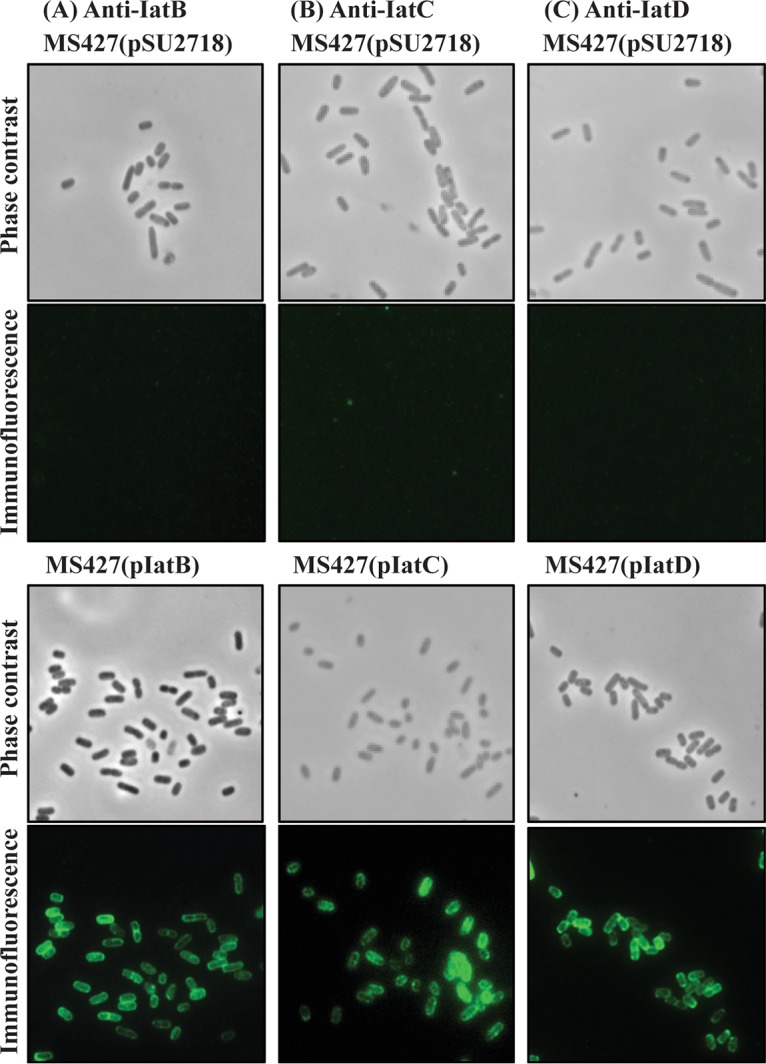
IatB, IatC, and IatD are localized at the cell surface when overexpressed in the MS427 background. Shown are images from phase-contrast and immunofluorescence microscopy using specific antisera against proteins IatB (A), IatC (B), and IatD (C). Positive reactions indicating the surface localization of IatB, IatC, and IatD were detected in MS427 (bottom) but not in the MS427(pSU2718) vector control (top).

To examine if the IAT proteins were localized to the outer membrane, immunofluorescence microscopy was performed using antisera specific to each protein. A strong fluorescence signal was observed for MS427(pIatB), MS427(pIatC), and MS427(pIatD), suggesting that these three proteins were effectively translocated to the cell surface in our recombinant E. coli strain (Fig. 2).

### Phenotypic properties of IatB, IatC, and IatD.

**FIG 3:**
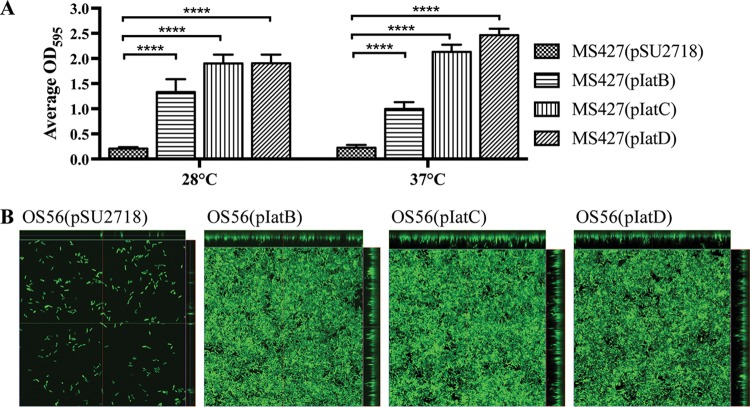
IatB, IatC, and IatD mediate biofilm formation. (A) Biofilm formation by E. coli strain MS427 in microtiter plates harboring plasmids pSU2718 (vector control), pIatB, pIatC, and pIatD. All strains were grown in M9 minimal medium in the presence of 1 mM IPTG to induce IAT protein expression, and plates were incubated at either 28°C or 37°C. Bar charts represent the average absorbance values at 595 nm, and error bars show the standard deviations calculated from three separate experiments (****, *P < *0.0001). (B) Confocal laser scanning microscopy images of biofilms formed on plastic coverslips under continuous-flow conditions 16 h after inoculation with OS56(pSU2718) (vector control), OS56(pIatB), OS56(pIatC), and OS56(pIatD). The images represent horizontal sections within each biofilm. Displayed at the top and right of each image are vertical sections representing the *xz* and *yz* planes, at the positions indicated by the green and red lines, respectively.

AT proteins are often associated with functions including cell aggregation, adhesion to extracellular matrix (ECM) proteins, and biofilm formation. While overexpression of the IatB, IatC, and IatD IAT proteins in MS427 did not lead to autoaggregation or adhesion to ECM proteins, all three IATs promoted strong biofilm formation. We first assessed this phenotype using a static microtiter plate biofilm assay, where MS427(pIatB), MS427(pIatC), and MS427(pIatD), but not the vector control strain MS427(pSU2718), were able to form a biofilm (Fig. 3A). The ability of the three IAT proteins to mediate biofilm formation was further explored using the *gfp*-tagged OS56 strain (a derivative of MS427) in a continuous-flow chamber setup, which permitted the distribution of cells within the biofilm to be monitored using confocal laser scanning microscopy. Consistent with the microtiter plate biofilm analyses, OS56(pIatB), OS56(pIatC), and OS56(pIatD) formed a significant biofilm with higher total biovolume, substratum coverage, and mean thickness (*P < *0.0001) than for the vector control strain after 16 h of growth in M9 minimal medium supplemented with 1 mM IPTG (Fig. 3B). These results demonstrate that IatB, IatC, and IatD are able to mediate biofilm formation when overexpressed in a recombinant K-12 background.

### Identification of genes involved in regulation of *iatB*.

Based on the high prevalence of *iatB* in E. coli phylogroup B2 (37/39 strains) (Fig. 1A), we selected this gene for further analysis and attempted to understand its regulation in SMS-3-5. The SMS-3-5 strain carries a large 130-kb plasmid (pSMS35_130; GenBank accession number CP000971.1) containing nine antibiotic resistance genes [*aadA2*, *aph(3′)-la*, *strA*, *strB*, *bla*_TEM-1B_, *catA2*, *sul2*, *tet*(A), and *drfA14*). To enable genetic manipulation of this strain, we first cured plasmid pSMS35_130 using a counterselectable vector strategy (29) to generate the strain SMS-3-5^c^.

**FIG 4:**
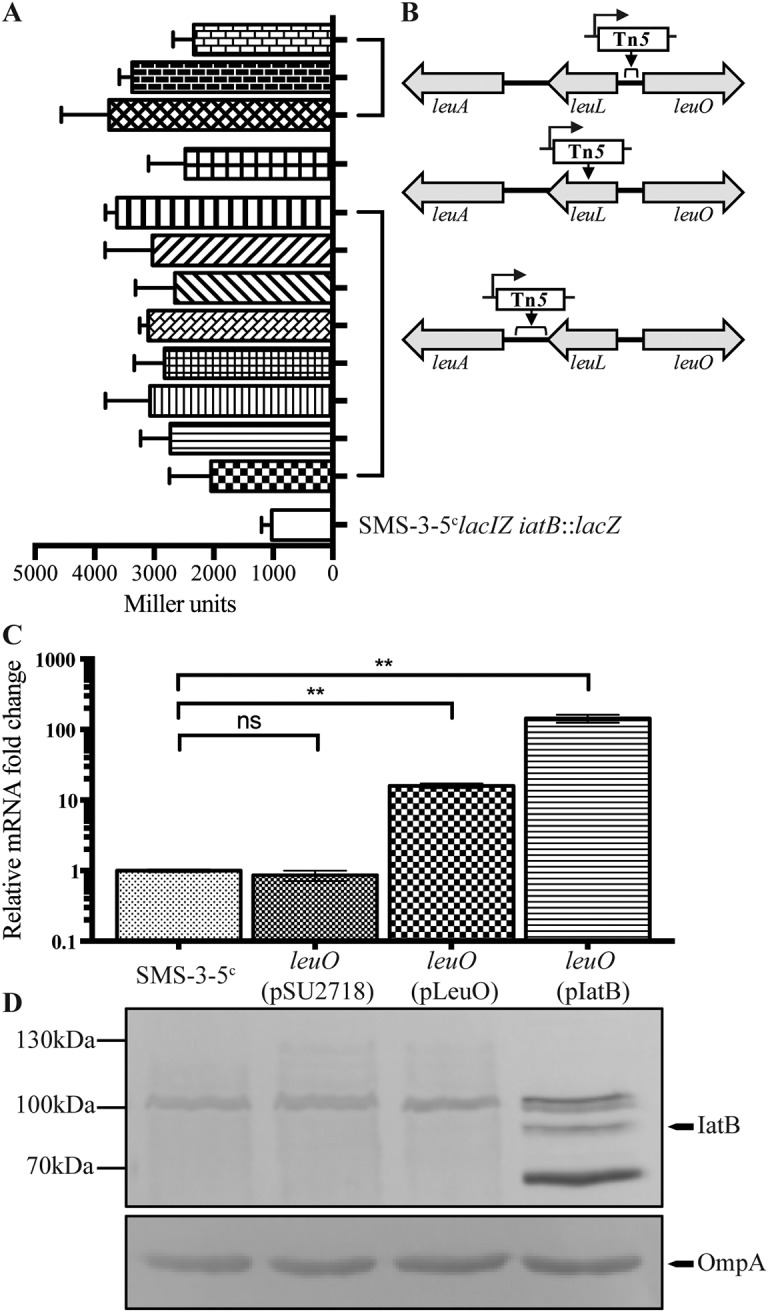
Effect of LeuO on *iatB* transcription in SMS-3-5^c^. (A) β-Galactosidase activity (in Miller units) of each mutant in comparison to the SMS35^c^*lacIZ iatB*::*lacZ* control strain. (B) Schematic diagram depicting the location and orientation of the Tn*5* insertions. (C) qRT-PCR showing the relative fold differences of *iatB* transcript levels in SMS-3-5^c^, SMS-3-5^c^*leuO*(pSU2718), SMS-3-5^c^*leuO*(pLeuO), and SMS-3-5^c^*leuO*(pIatB) (**, *P = *0.0069 to 0.009; ns, not significant). (D) Western blot analysis of IatB in the respective strains. A band corresponding to IatB was detected only in the SMS-3-5^c^*leuO*(pIatB) strain.

To investigate the genetic basis of *iatB* regulation, we generated a chromosomal *iatB* promoter-*lacZ* reporter fusion strain (SMS-3-5^c^*lacIZ iatB*::*lacZ*). SMS-3-5^c^*lacIZ iatB*::*lacZ* was subjected to transposon mutagenesis using a mini-Tn*5* cassette, generating approximately 30,000 transposon mutants that were screened for blue color development on lysogeny broth (LB) plates containing 5-bromo-4-chloro-3-indolyl-β-d-galactopyranoside (X-gal). In this screen, we identified 12 colonies that were dark blue, indicating increased *iatB* promoter activity, and this was confirmed by their increased β-galactosidase activity compared to that of the SMS-3-5^c^*lacIZ iatB*::*lacZ* parent strain (Fig. 4A). Further analysis revealed that the mutants all contained independent insertions within three regions of the *leu* operon: (i) three insertions in an intergenic region between the *leuL* and *leuO* genes, (ii) one insertion within the *leuL* gene, and (iii) eight insertions in an intergenic region between the *leuL* and *leuA* genes (Fig. 4B). Detailed analysis of the 12 Tn*5* insertions revealed that they were all oriented in the same direction, with the chloramphenicol resistance gene placed in the same orientation as the downstream *leuO* gene. In previous studies, we have shown that the promoter of the chloramphenicol resistance gene in this Tn*5* transposon can drive the transcription of a downstream gene if the insertion position is favorable (30, 31). Hence, we hypothesized that the Tn*5* insertions caused increased transcription of the *leuO* gene, which in turn resulted in enhanced *iatB* promoter activity.

### *iatB* mRNA transcript levels are affected by the global regulator LeuO.

To directly examine the effect of LeuO on *iatB* expression, the *leuO* gene was mutated in SMS-3-5^c^ to generate strain SMS-3-5^c^*leuO*. In addition, the *leuO* gene was also PCR amplified, cloned into the expression vector pSU2718 (to generate the plasmid pLeuO), and transformed into the SMS-3-5^c^*leuO* mutant. The expression of *iatB* was first examined in SMS-3-5^c^, SMS-3-5^c^*leuO*(pSU2718), SMS-3-5^c^*leuO*(pLeuO), and SMS-3-5^c^*leuO*(pIatB) by quantitative reverse transcription PCR (qRT-PCR) (Fig. 4C). In this assay, overexpression of *leuO* led to an ∼15-fold increase in relative *iatB* transcript levels, whereas *iatB* transcript levels in the SMS-3-5^c^*leuO*(pSU2718) vector control strain were similar to those in wild-type SMS-3-5^c^. In comparison, the level of the *iatB* transcript in SMS-3-5^c^(pIatB) was ∼144-fold higher than in SMS-3-5^c^. Next, the expression of IatB was examined by direct detection of the protein using Western blotting (Fig. 4D). Although the IatB protein was clearly detected in SMS-3-5^c^(pIatB), it was not detected in any of the other strains. Taken together, these results suggest that overexpression of *leuO* increases *iatB* transcript levels in SMS-3-5^c^, but this increase in transcription does not translate into detectable levels of the IatB protein under the experimental conditions employed in this study.

## DISCUSSION

Proteins secreted by the type V secretion system exhibit extensive diversity, and we show here that this variation extends to IATs in E. coli. The three IAT proteins characterized in this study, IatB, IatC, and IatD, all promoted biofilm formation, suggesting that they may contribute to surface colonization under certain environmental conditions. By focusing on the regulation of *iatB*, which displayed a high prevalence in phylogroup B2 strains frequently associated with extraintestinal infection, we also identified LeuO as a putative regulator of IatB.

The presence of a short C-terminal passenger domain in IAT proteins, as observed for IatA, is not uncommon. IAT proteins are almost exclusively found among the *Gammaproteobacteria*, and many IATs possess short C-terminal extensions that share structural characteristics with bacterial immunoglobulin superfamily (IgSF) domains (e.g., a fibronectin III domain) (24, 32). These domains can participate in protein-protein interactions and have been identified in factors that mediate the translocation of protein substrates across the outer membrane (32). Modeling of the IatA passenger domain in Phyre2 suggests that it possesses structural characteristics similar to those of a fibronectin III domain (data not shown). IatA is also the most common IAT protein of E. coli, and by analogy to other proteins that contain IgSF domains, it is possible that IatA could assist in the translocation of other proteins across the outer membrane.

IatB is an orthologue of SinH from *Salmonella* and shares 74% amino acid identity. Analysis of this locus revealed that the coding sequences immediately downstream of *iatB* (i.e., EcSMS35_2660) and *sinH* (i.e., *sinI*) also share sequence similarity and encode predicted proteins that lack a β-barrel domain. These gene clusters are analogous to the well-characterized *zirTSU* operon found in *Salmonella*. The ZirT protein shares a similar domain organization with SinH and IatB: it contains an N-terminal IAT β-barrel domain and a C-terminal passenger containing IgSF domains. In addition, ZirT serves as a platform for the translocation of ZirS and ZirU (33, 34), and thus, it is possible that IatB (and SinH) could possess similar functional properties. Our results demonstrate that IatB is able to mediate biofilm formation independent of the downstream EcSMS35_2660 protein. However, whether IatB also acts as a transport platform for other proteins remains an intriguing subject for further investigation.

The typical IAT passenger domain contains variable repeats of Big1 domains with a similar fold despite their low sequence similarity (17, 27). Of the four new IAT proteins identified in this study, IatB and IatC contain passenger domains of various lengths. The passenger domains of some IATs, such as the invasin-like molecule of Yersinia ruckeri, contain Ig domains that are almost identical (35). The presence of tandem sequence repeats can result in misassembly of a gene sequence due to erroneous sequencing analyses (36). However, we found no evidence to support the idea that there is variability in the number of tandem repeats in IatC or IatD. Other AT proteins, like UpaH and Ag43, exhibit sequence variation that results in altered levels of biofilm formation by different variants (13, 37). Thus, it is possible that different variants of IatB and IatC may possess different functional properties, such as various degrees of biofilm formation.

We showed that IatB, IatC, and IatD were able to mediate biofilm formation when expressed in a recombinant K-12 strain. Like intimin and invasin, bioinformatic analysis predicts that the passenger domains of IatC and IatD are capped with a C-type lectin domain. The C-type lectin domain of intimin mediates adhesion of enteropathogenic E. coli strains to the intestinal epithelium via interaction with its receptor Tir, whereas the domain found in invasin mediates binding to β_1_-integrins. Thus, it is possible that the C-type lectin domains of IatC and IatD could also recognize specific receptors on host surfaces that remain to be identified. The IatC and IatD proteins also contain a LysM domain, which has also been identified in other large IAT proteins (26, 38). The LysM domain is found in many peptidoglycan-binding proteins and may contribute to their localization and stability in the outer membrane (38, 39).

We also sought to understand the regulation of the *iatB* gene. The regulation of AT protein expression in E. coli is complex; for example, many AIDA-I AT proteins are not expressed during standard laboratory growth, and multiple control mechanisms have been described (7, 8, 40). Consistent with this, we were unable to detect expression of the IAT proteins in wild-type SMS-3-5 via Western blot analysis following static or shaking growth in either LB or M9 minimal medium at 28°C, 37°C, or 42°C (data not shown). Using a mutagenesis approach, we identified LeuO as a potential activator of *iatB*. However, while overexpression of LeuO led to an increase in the *iatB* transcript in SMS-3-5^c^, direct expression of the protein could not be detected via Western blotting. This may have been due to its low level of expression or instability or even the lack of additional regulatory factors that are required for its optimal expression. LeuO is a LysR-type transcriptional regulator (LTTR) that contains an N-terminal helix-turn-helix DNA-binding domain (41) and can be found in other members of the *Enterobacteriaceae*, including *Salmonella*, *Shigella*, and *Yersinia* spp. (41). LeuO is involved in coordination of the bacterial stress response, and expression of LeuO is increased upon entry into stationary phase (42–44). Additionally, LeuO activates several cryptic fimbriae of E. coli, and overexpression of LeuO in E. coli led to increased cell adhesion and biofilm formation (42). Our findings are consistent with these phenotypic properties, suggesting that further work is now required to understand the molecular mechanisms by which LeuO controls IatB expression and to determine if LeuO plays a role in the coordinated regulation of other IATs.

Overall, this study has identified the complement of IAT proteins present in E. coli and provides new insight into their diversity, function, and regulation. Four of these IAT proteins were new, and three were functionally characterized. We found that the IATs IatB, IatC, and IatD are surface exposed, mediate biofilm formation, and thus may comprise part of the arsenal of factors used by E. coli to colonize different surfaces. Further work is now needed to understand the molecular mechanisms that control their expression.

## MATERIALS AND METHODS

### Bioinformatics analysis.

The E. coli database was represented by 126 published complete genomes available in the NCBI database. Sequence comparisons were examined using the fasta36 software package (45). The database of annotated proteins from each strain was generated using the cds_extractor v0.7.1 tool (46) and probed using fasta36 to identify putative β-barrel domain-containing IAT proteins. The context of the β-barrel domain was examined in each extracted protein sequence to ensure that it was located at the N-terminal end of the protein. The prevalence of genes was determined using tfastx36 and a cutoff of >75% identity over an 80% amino acid sequence alignment. Any proteins lacking an N-terminal signal sequence or the β-barrel domain were discarded. Multilocus sequence typing (MLST) analysis was performed using the sequences of seven housekeeping genes as previously described (47). The E. coli strains were classified into major phylogroups (A, B1, B2, D, E, and F) as previously described (48). Briefly, strains were sorted into the different phylogroups based on an *in silico* analysis of the *arpA*, *chuA*, *yjaA*, and TSPE4.C2 loci using fasta36 with a cutoff of >90% identity over a 95% nucleotide sequence alignment. The genomic context of genes was analyzed and drawn with Easyfig (49). Alignments were constructed in MEGA7 (50) using the Muscle algorithm with default settings. Trees were produced with MEGA7 using the maximum likelihood method with default settings and supported with 100 bootstraps. The Conserved Domain Database (CDD) (51), InterPro (52), and Phyre2 (53) were used to analyze protein structures, and SignalP4.1 (54) was used to predict the presence of signal sequences.

### Bacterial strains and culture conditions.

Strains were routinely cultured on solid or in liquid lysogeny broth (LB) or M9 minimal medium and supplemented with the following antibiotics where appropriate: gentamicin (Gent) (20 μg/ml), ampicillin (Amp) (100 μg/ml), kanamycin (Kan) (50 μg/ml), and chloramphenicol (Cm, 30 μg/ml). Expression of genes was induced with either 1 mM isopropyl-β-d-1-thiogalactopyranoside (IPTG) or 0.2% l-arabinose when required. The bacterial strains and plasmids used in this study are outlined in Data Set S3 in the supplemental material.

### Molecular methods.

Methods for DNA extraction, purification, sequencing, and PCR were performed as previously described (8, 55, 56). Deletion mutants were constructed using a modified λ red recombinase gene replacement system as described previously (57–59). For RNA extraction, exponentially growing cells grown in LB (500 μl) (optical density at 600 nm [OD_600_] = 0.6) were stabilized in 1 ml of RNAprotect bacterial reagent (Qiagen). Subsequent RNA extraction, DNase I treatment, first-strand cDNA synthesis, and qRT-PCR were performed as previously described (60). Gene expression levels were determined with the 2^−ΔΔ*C_T_*^ method (61), with relative fold differences expressed against wild-type SMS-3-5. All experiments were performed in three independent replicates. RT-PCRs were performed with primers specific for each gene. The full list of primers used is shown in Data Set S4.

10.1128/mSphere.00572-19.8 DATA SET S4 Primers used in this study. Download Data Set S4, XLSX file, 0.1 MB. Copyright © 2019 Goh et al. 2019 Goh et al. This content is distributed under the terms of the Creative Commons Attribution 4.0 International license.

### Generation of polyclonal antibodies.

A fragment of the *iatA-D* genes corresponding to the C-terminal passenger domain was PCR amplified from SMS-3-5, cloned as an N-terminal 6×His fusion protein in plasmid pMCSG7 via ligation-independent cloning (LIC) (62), and transformed into E. coli BL21(DE3). The expression of each of the 6×His-tagged fusion proteins was induced with 1 mM IPTG and purified on a Ni-nitrilotriacetic acid (NTA) spin column (Qiagen). The purified proteins were quantified with a bicinchoninic acid protein assay kit (Sigma) and assessed for purity via SDS-PAGE. These purified proteins were used to generate polyclonal antisera in rabbits at the Walter and Eliza Hall Institute of Medical Research Antibody Facility.

### Protein sample preparation and immunoblotting.

Whole-cell lysates were prepared by pelleting 1 ml of OD_600_-standardized cells and resuspending the cells in 50 μl water and 50 μl 2× SDS loading buffer (100 mM Tris-HCl, 4% [wt/vol] SDS, 20% [vol/vol] glycerol, 0.2% [wt/vol] bromophenol blue [pH 6.8]). SDS-PAGE and transfer of proteins onto polyvinylidene difluoride (PVDF) membranes for Western blot analysis were performed as previously described (63). Polyclonal antisera specific for each protein were used to probe for the respective proteins, and alkaline phosphatase-conjugated anti-rabbit antisera (Sigma-Aldrich) were used as the secondary antibodies.

### Immunofluorescence.

Cultures grown overnight and supplemented with the appropriate antibiotics and 1 mM IPTG were fixed to an OD_600_ of 0.4, spotted onto a glass slide, and allowed to dry. The cells were fixed with 4% paraformaldehyde (PFA), blocked with 0.5% bovine serum albumin (BSA), and incubated with a 1:100 dilution of the appropriate primary antibody in phosphate-buffered saline (PBS) for 30 min. The cells were washed and incubated with secondary goat anti-rabbit antiserum coupled to fluorescein isothiocyanate (FITC) diluted 1:500 in PBS. The slides were washed and air dried, mounted with Prolong gold antifade reagent (Life Technologies), and examined under a Zeiss Axioplan 2 epifluorescence light microscope.

### Phenotypic assays.

Biofilm assays were performed in 96-well PVC plates (Corning) as previously described (64). Statistical analyses were performed using unpaired two-tailed Student’s *t* test. Flow cell experiments were performed as previously described (65, 66). Biofilm thickness, coverage, and total biomass measurements were collected from 10 z-stacks for each strain and analyzed with the COMSTAT program (67). The nonparametric Kruskal-Wallis test within GraphPad Prism 7 software was used for statistical analysis; *P* values of <0.05 were considered significant. β-Galactosidase assays were performed as previously described (68). Each strain was assessed in quadruplicate, and experiments were performed in two independent replicates.

### Transposon mutagenesis.

Transposon mutagenesis of SMS-3-5*lacIZ iatB*::*lacZ* was performed using the Epicenter EZ::Tn*5* transposome construction kit as previously described (60). The transposon insertion site of the mutants was identified via 2-step arbitrary PCR as previously described (69).

### Curing of plasmid pSMS35_130.

Curing of the pSMS35_130 plasmid was performed as previously described (29). Briefly, the incompatibility regions (IncFIA and IncFII) and antitoxins (*sok*, *vagC*, and *pemI*) from pSMS35_130 were synthesized (Epoch Life Science Inc.) and incorporated directly into plasmid pMDP4, which contained a chloramphenicol resistance gene cassette, the *gfp* gene, and the *sacB* gene. This plasmid is referred to as pSMS35_130cure. Plasmid pSMS35_130cure was electroporated into SMS-3-5, and transformants were plated on LB agar containing chloramphenicol. Colonies were screened for fluorescence indicating the presence of the pSMS35_130cure plasmid. After one round of subculture, cells were plated onto LB agar containing 5% sucrose for subsequent selection of plasmid-free cells. Plasmid loss was confirmed by antibiotic sensitivity testing.
