# Supplementary figures and images for: Bioinformatic and Molecular Analysis of Inverse Autotransporters from Escherichia coli
Source: mSphere. 2019 Aug 28;4(4):e00572-19. doi: 10.1128/mSphere.00572-19 (PMC6714894; doi:10.1128/mSphere.00572-19)

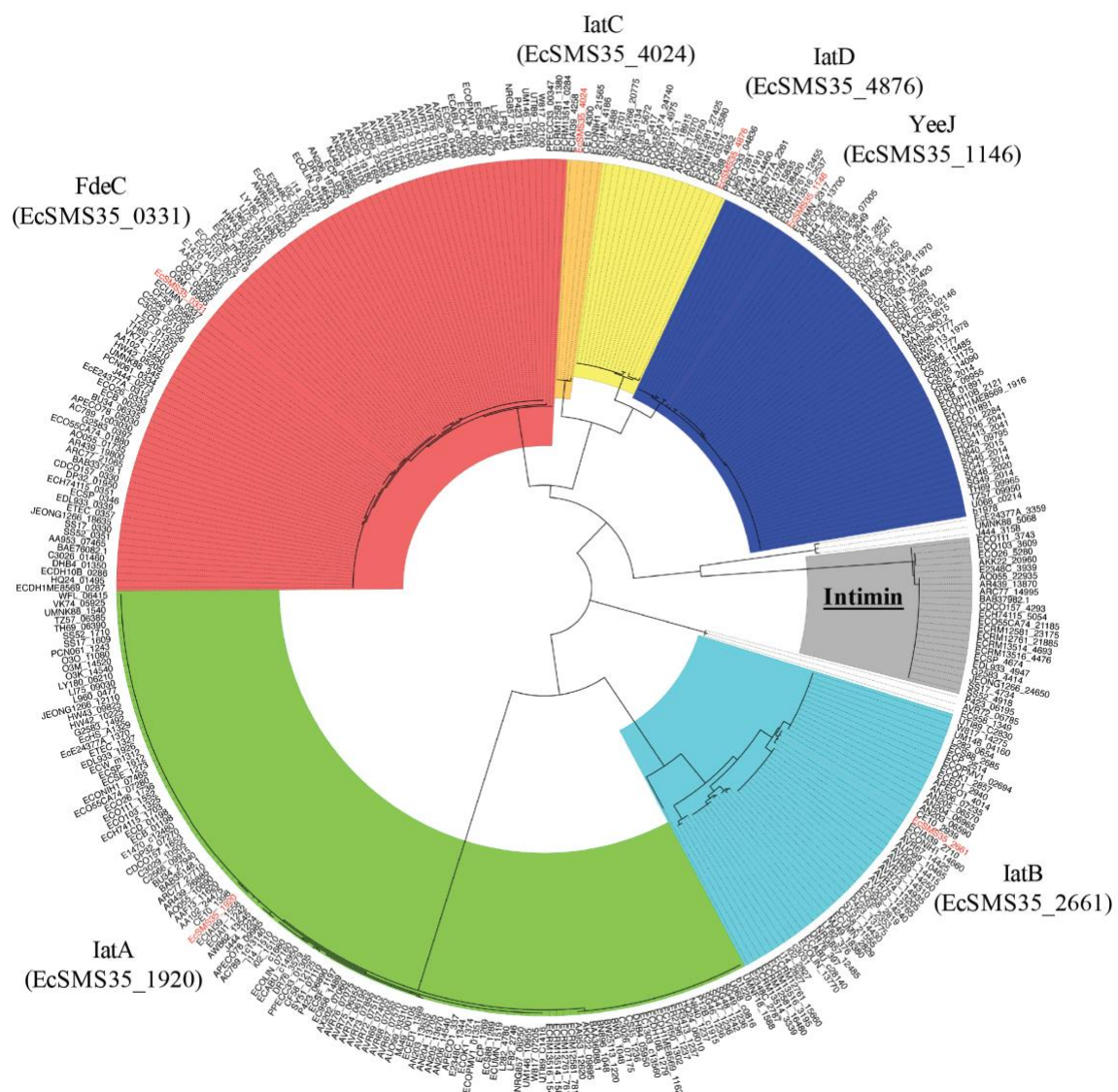

**Figure S1**

Supplement: FIG S1 [file mSphere.00572-19-sf001.pdf]

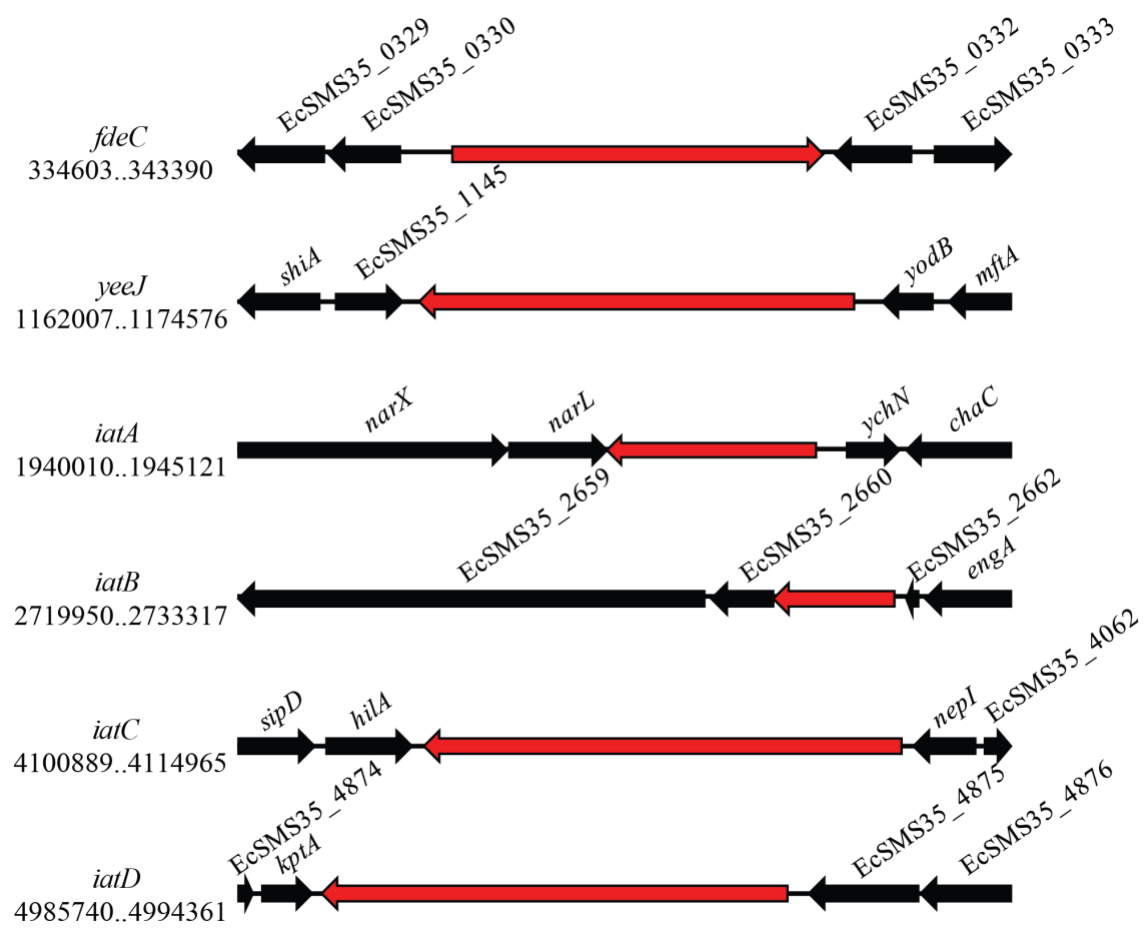

**Figure S2**

Supplement: FIG S2 [file mSphere.00572-19-sf002.pdf]

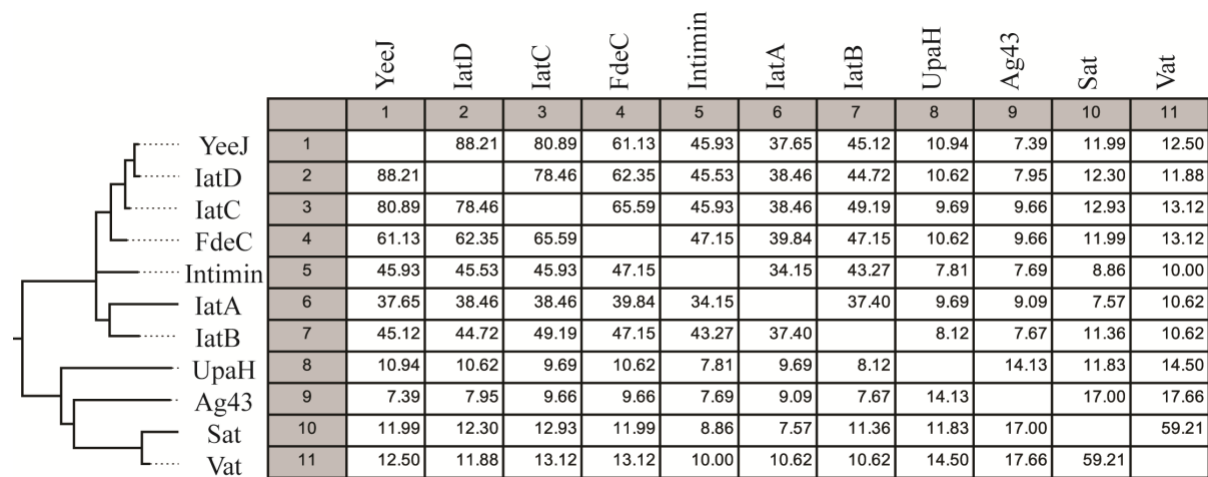

**Figure S3**

Supplement: FIG S3 [file mSphere.00572-19-sf003.pdf]

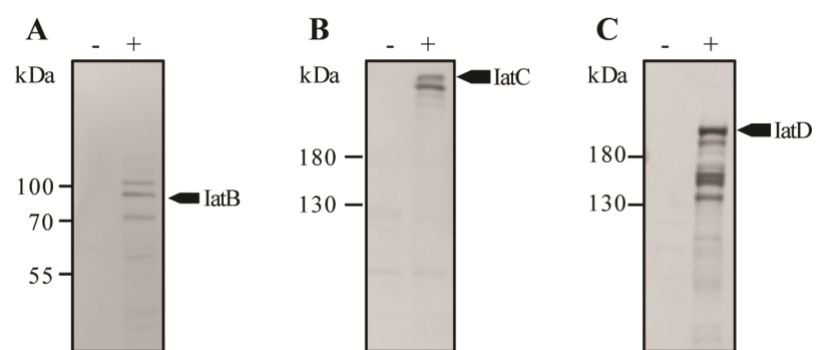

**Figure S4**

Supplement: FIG S4 [file mSphere.00572-19-sf004.pdf]
